# Volar plating: functional recovery of the pronator quadratus

**DOI:** 10.1007/s00590-021-03133-7

**Published:** 2021-09-30

**Authors:** Patrick Porter, Alasdair MacInnes, Tim Drew, Weijie Wang, Rami Abboud, Graeme Nicol

**Affiliations:** 1 Department of Orthopaedic and Trauma Surgery, Ninewells Hospital and Medical School, University of Dundee, Dundee, Scotland, UK; 2 Dean’s Office Faculty of Engineering, Al Koura Campus, University of Balamand, El-Koura, Lebanon

**Keywords:** Pronator quadratus, Pronator teres, Forearm pronation, Torque, Grip strength

## Abstract

**Purpose:**

The pronator quadratus (PQ) is reflected in the surgical approach to the distal radius. This study explores the functional strength of PQ, 12 months after volar plating without repair of PQ.

**Methods:**

A total of 135 patients were identified from our prospectively collected database. All volunteers had grip strength and pronation power tested in the treated and contralateral forearms at 45, 90 and 135 degrees of elbow flexion using a custom-built torque measuring device and hydraulic hand dynamometer to evaluate forearm pronation.

**Results:**

Twenty-seven participants were included in the study. No significant difference was identified in mean peak pronation torque between the volar plated and non-treated forearms. Pronation strength was identified as being independent of angle of elbow flexion. Grip strength was correlated with forearm pronation showing no significant difference between groups.

**Conclusions:**

Our results suggest adequate long-term (15–32 months) functional recovery of the pronator quadratus after volar plating.

**Level of Evidence:**

III.

## Introduction

Fractures of the distal radius account for approximately 18% of all adult fractures globally and are one of the most common injuries presenting as orthopaedic trauma [1, 2]. An increase has been identified over recent decades which positively correlates with a more physically active ageing population [2–4]. There are a variety of methods used to treat distal radius fractures including manipulation under anaesthetic, casting, Kirschner-wire (*K*-wire) fixation, external fixation and volar plating. Patients who sustain a volar displaced fracture or an unstable fracture pattern have been found to have an increased risk of post-traumatic arthritis, deformity and pain if managed with manipulation and casting alone. Therefore, these patients are often managed with surgical fixation. There is debate in the literature as to the optimum surgical fixation, and there has been a recent trend towards the less invasive option of *K*-wire fixation [5]. However, volar plating remains a widely used surgical option and has been shown to have a slightly better Patient Rated Outcome Score (PROMs) at 3 months. The benefits of volar plating are immediate fracture stabilisation, lower mal-union rates and early recovery [5, 6].

Volar plating is typically performed using a *Flexor Carpi Radialis* approach: deep dissection involves the release of the superficial head of the *pronator quadratus* (PQ) which is the predominant muscle of forearm pronation [7–9]. The PQ is located at the distal radius and is comprised of a superficial and deep head both of which generate torque to carry out forearm pronation. The PQ is a muscle with a broad musculotendinous insertion making it difficult to reattach after volar plating, due to the integrity of the muscle and the tension required to repair the muscle once the plate has been attached. A survey carried out by Swigart et al. 2012 reported that up to 27% of American orthopaedic surgeons do not formally reattach the PQ but simply lay the muscle over the top of the plate to avoid tendon aggravation [10]. In the UK, surgeons do likewise, but no literature exists on PQ reattachments. It has been considered that repair of the PQ acts to protect the tendons and improve post-operative recovery.

An important part of rehabilitation is assessing global wrist function post-operatively. This is usually undertaken during routine follow-up at 6 weeks post-surgery. Routinely this will involve clinical examination and patient feedback. However, there is no objective measure that can be used to evaluate muscle recovery. Previous studies have measured pronation strength in patients following volar plating up to 12 months post-operatively [11–13]. This study aimed at evaluating pronation torque and grip strength in patients who have been treated with volar plates beyond 12 months. This was undertaken by assessing the relevance of the surgical incision to the PQ in patients who underwent a distal radius fracture, repaired with a volar plate and without repair of the PQ muscle. The functional recovery of the PQ in these patients was evaluated by measuring pronation torque and grip strength and comparing these outcomes with patients’ non-injured, non-treated contralateral forearms.

## Methods

### Ethical approval

The study was conducted in a University teaching hospital with prior approval of the Health Research Authority (REC reference 18/ES/0004 (17/ES/0162)). A total of 135 patients with distal radius fractures were retrospectively identified via the Picture Archiving and Communication System between January 2014 and February 2016.

### Inclusion and exclusion criteria

**Fig. 1:**
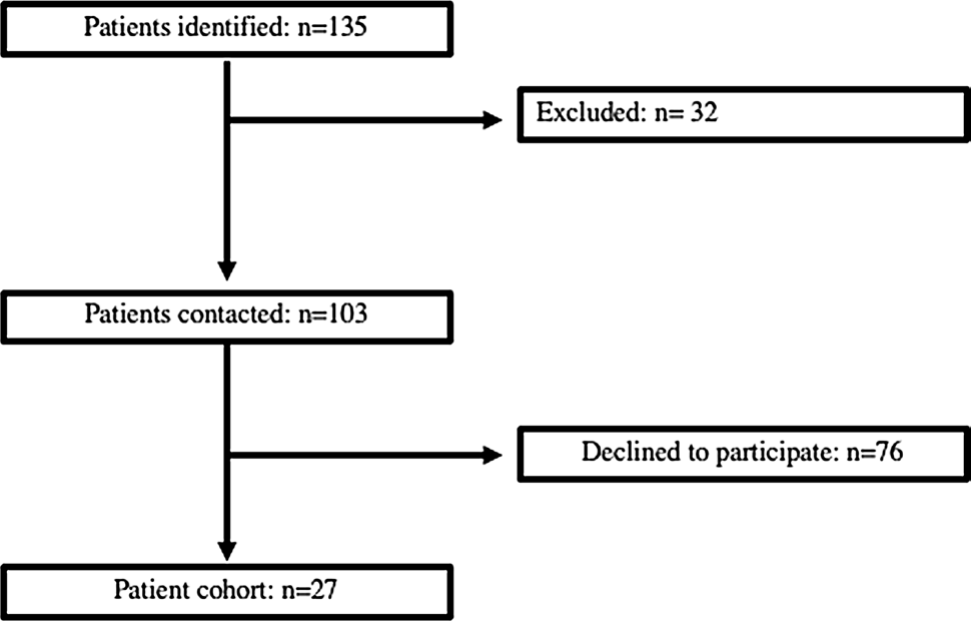
Flowchart describing the process by which patients were identified to participate in the study

Those who met the inclusion criteria were defined as patients who were treated with volar locking plates, discharged from clinic, 12-month post-operation and sustained a type I, II, III, IV, V or VI Frykman fracture. Those who met the exclusion criteria were categorized as having previous history of significant wrist or upper limb injury apart from the known distal radial fracture, bilateral wrist fracture, Frykman VII and VIII fractures and concurrently suffered from musculoskeletal, neuromuscular or spinal disease. After the application of the exclusion criteria, a total of 103 patients were contacted and 27 voluntarily took part in the study (Fig. 1). The patients had been treated by multiple surgeons within the same hospital trust using similar volar locking plates (DVR-Zimmer Biomet, Warsaw Indiana, USA), and all PQs were unrepaired.

### Assessment

**Fig. 2:**
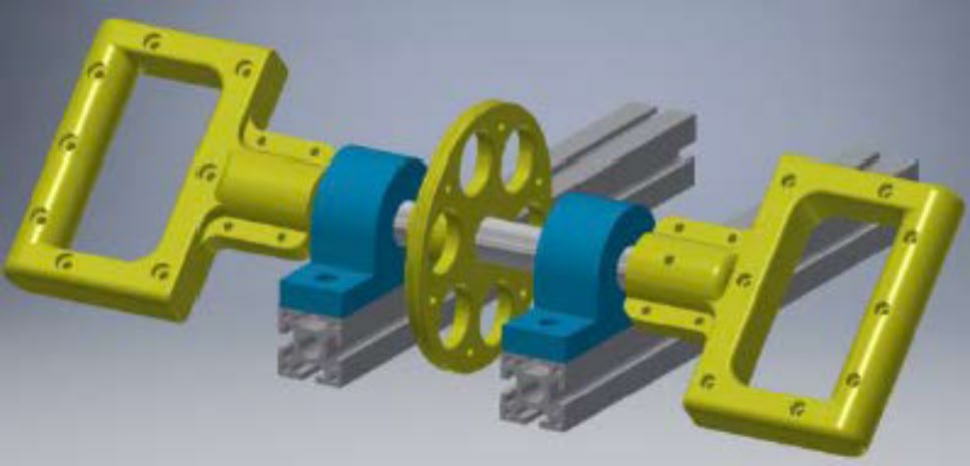
Computer-aided design of the purpose-built device. The device was designed to permit a resistant pronation effort allowing for torque generated to increase linearly with angular displacement

**Fig. 3:**
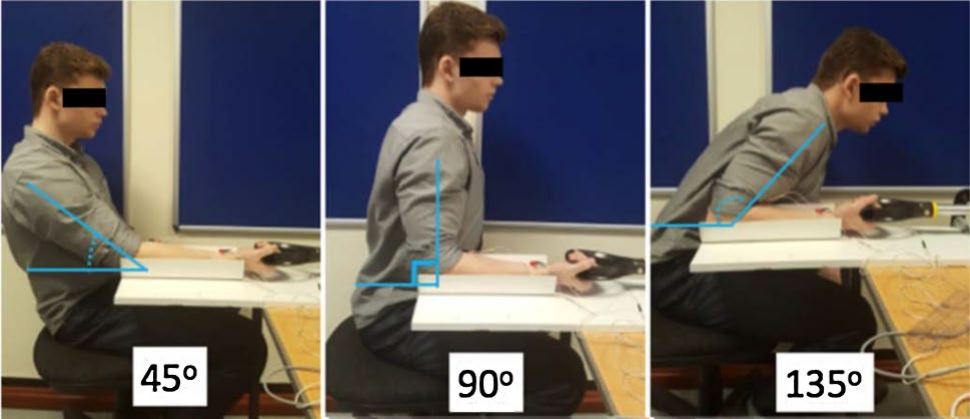
Participant pronating at varied angles of elbow flexion. The shoulder was placed in neutral position and the elbow flexed at either 45, 90, 135 degrees during each pronation trail

Pronation strength was recorded using custom written program (Software version 1.2, UDOTs, Scotland) on a purpose-built device (Fig. 2). The custom written program and the purpose-built device had been previously verified on a cohort of 32 uninjured volunteers [14]. Data were recorded in varying degrees of elbow flexion, emulating real-life functional positioning to best isolate the PQ muscle contribution to forearm pronation. With the shoulder in neutral position and the elbow flexed at either 45, 90, 135 degrees, the forearm was pronated with three trials while recording minimum and maximum peak torque of every trail (Fig. 3). Each forearm (treated and non-treated) underwent pronation testing at each of the angle of elbow flexion (45, 90, 135 degrees) in a randomised fashion. The mean peak torque value was calculated for each angle of elbow flexion for each forearm.

Maximum grip strength was recorded manually using a JAMAR® Hydraulic hand dynamometer with the elbow at 90° elbow flexion and shoulder in neutral position. The measurements were repeated three times in each forearm. The mean grip strength was calculated and converted into grip strength ratio in accordance with Bremur et al. 2014 [15].

### Statistical analysis

Statistical analysis was carried out using SPSS statistical software 13.0. Data were presented as mean and median to the underlying distribution. Continuous variables were described as mean and ± standard deviation (SD) or range. Paired data were analysed with a pairwise comparison test. The *P* value for statistical significance was set as < 0.05.

## Results

The final study group consisted of 27 patients with 23 women, 4 men and a mean time since surgery of 25 months (range 15–32 months). The mean age of the cohort was 59 years (range 21–71). The majority of the cohort was aged under 65 and 33% aged 65 to 71. There was an even split of dominant and non-dominant forearms treated with volar plating. The cohort grip strength ratio (treated/non-treated) was calculated at 0.98. The baseline patient characteristics are displayed in Table 1.

**Table 1:** Baseline patient characteristics expressed as mean (range) or *n*

*n* (female/male)	Age (years)	Mean post-operation (months)	Frykman classification		Volar plated forearm		Cohort grip strength	
27 (23/4)	58.81 (21–71)	25 (15–32)	I II III IV V VI	3 9 4 6 1 4	Dominant	13	Mean treated (kg)	23.67
					Non-dominant	14	Mean non-treated (kg)	24.47
							Grip strength ratio (SD)	0.98 (± 0.055)

### Torque

**Fig. 4:**
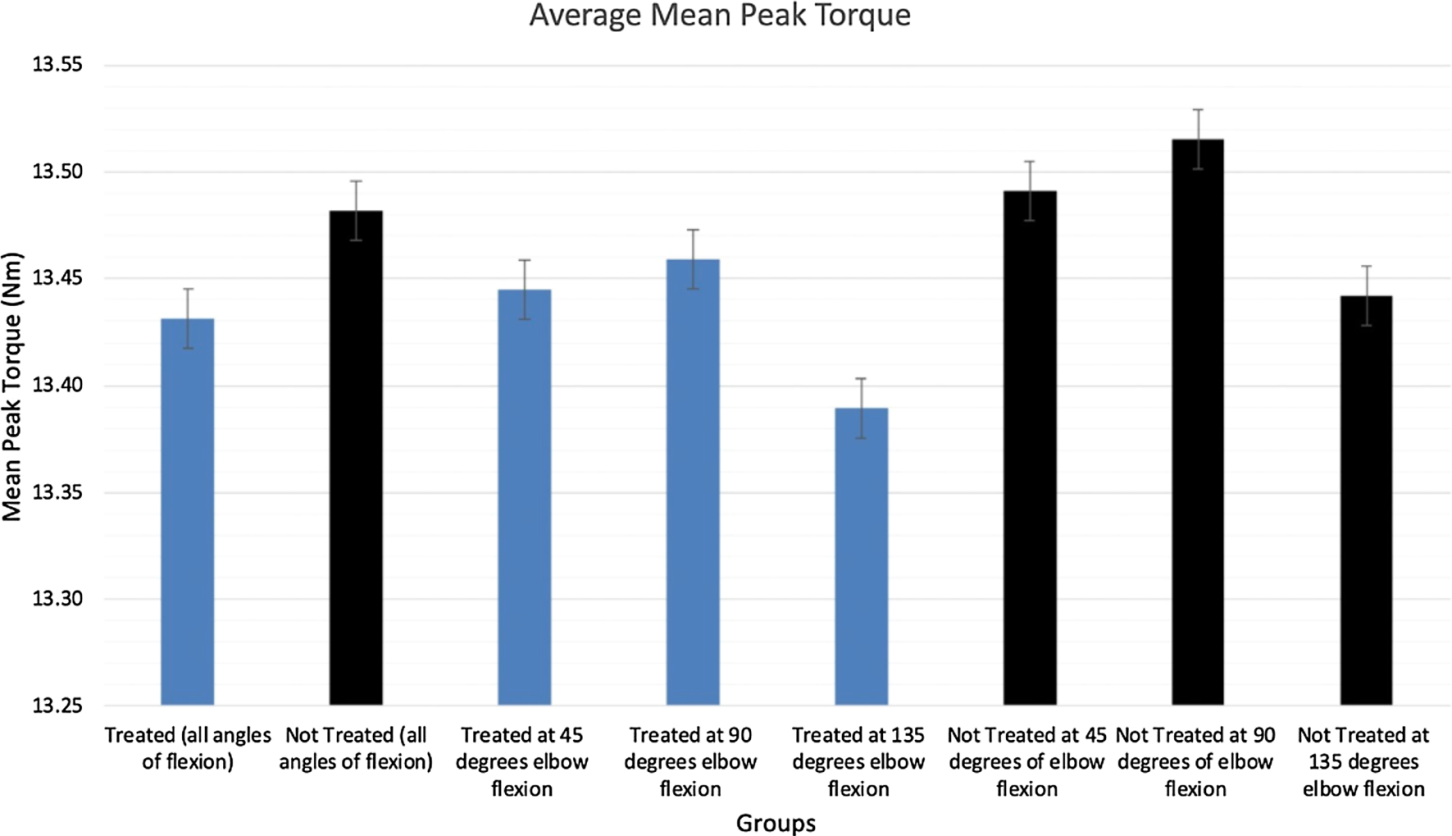
Mean peak torque in treated and non-treated forearms at the three variations of elbow flexion at 45, 90 and 135 degrees

There was a non-significant trend in both treated and non-treated forearms for mean peak torque to be greatest at 90° elbow flexion followed by 45° and then 135° elbow flexion (Fig. 4). Pairwise comparison of mean peak torque was not found to be statistically significantly different between treated and non-treated forearms (13.48 Nm Vs. 13.43 Nm) at all angles of elbow flexion (Table 2), indicating that elbow flexion is independent of peak torque (Table 2).

**Table 2:** Pairwise comparisons of mean peak torque between treated and non-treated forearms at 45, 90 and 135 degrees of elbow flexion

Pairwise comparisons between treated and non-treated forearms					
Angle of elbow flexion	Mean difference	Std. error	Significance	95% Confidence interval for difference	
				Lower bound	Upper bound
All (45, 90, 135)	0.050	0.035	0.165	− 0.022	0.123
45	0.047	0.037	0.222	− 0.030	0.123
90	0.056	0.036	0.129	− 0.017	0.130
135	0.053	0.045	0.254	− 0.040	0.146

### Grip strength

Grip strength was found to be well correlated with pronation strength at all angles of elbow flexion in both treated and non-treated forearms (Table 3).

**Table 3:** Pearson correlation between treated and non-treated forearms at 45, 90 and 135 degrees of elbow flexion

Grip Strength	Angle of flexion (degrees)			
	All angles	45	90	135
Treated				
Pearson correlation	0.657	0.649	0.657	0.591
Significance	*P* < 0.0001	*P* < 0.0001	*P* < 0.0001	*P* < 0.0001
Non-treated				
Pearson correlation	0.672	0.631	0.624	0.704
Significance	*P* < 0.0001	*P* < 0.001	*P* < 0.001	*P* < 0.001

## Discussion

### Treated versus non-treated

There was no statistical difference between treated and non-treated forearms for pronation torque at all angles of elbow flexion. Our results suggest that volar plating, without repairing the PQ, does not result in a reduction (in mean peak torque) for pronation strength.

Pronation is collectively relied upon by the PQ and *pronator teres* (PT) as both muscles work to pronate the forearm [12, 16]. However, the larger superficial PQ is thought to generate the greatest amount of torque, independent of elbow flexion, and so is the predominant forearm pronation muscle [9]. In Mccrosky et al.’s prospective study, lidocaine, a local anaesthetic, was injected into the PQ, thus inactivating its contribution in the pronation effort. Results revealed there was a 21% reduction in pronation power at 90° of elbow flexion and 0° shoulder abduction compared to the contralateral non-treated forearm in a cohort of normal healthy individuals [17].

Our expectation was to see a diminution in pronation torque at 135° of elbow flexion due to the reported biomechanical disadvantage in which the PT is placed. The PT is the secondary muscle of forearm pronation and has a humeral origin that crosses the elbow joint [8]. Increasing the degree of elbow flexion would be expected to reduce the moment arm of the muscle. In order to address the potential issue of the diminution in pronation strength caused by the PT, the custom-built device was designed to mitigate this issue by increasing the activity of the PT. According to Basmajian et al., the relative activation of the PT is dependent upon the load type. Under a load of increasing resistance, the PT is significantly activated and reinforces in the pronation effort [9]. The custom-built device was designed for torque to increase linearly with angular displacement. This will have caused the pronation effort to increase in resistance over time. It should have resulted in the PT reinforcing the PQ over the duration of the pronation effort. Our study mimicked real-life situations and so is suggestive of a functional pronation effort.

The findings in this study are in keeping with the current literature; however, it is the first study to show that grip strength and forearm strength are maintained past 12 months post-operatively. An isokinetic study by Huh et al. [12] which did not vary the angle of elbow flexion found no significant difference in pronation strength in those treated with volar plating for a distal radial fracture at the 12-month post-operative period. The study analysed the isokinetic measurements of 33 patients at 6 months, and at 12 months. The results were directly compared to the patients’ non-treated hand and the mean forearm pronation peak torque of 17 healthy individuals from a previous study (McConkey et al.) [17]. The treated to non-treated recovery percentage ((treated/non-treated) *100) for Huh et al. was 92% at the 12-month period; in our study, the recovery percentage for our cohort was 99%. This shows a linear progression in the recovery of pronation strength between a cohort measured at 12 months and one measured with a mean post-operative time of 24 months. Huh et al. [12] reported that the PQ was not entirely repaired, but absorbable sutures were applied to the PQ at its radial border, which makes our results difficult to compare with their results.

### Grip strength in relation to global wrist function

Global wrist function is evaluated in this study. To account for pain or disability that may otherwise have prevented proper adherence to study procedures, grip strength was measured. The function of the PQ is vital as it plays an important role in both grip and pronation strengths. Shin et al.’s sonographic study identified the muscle activity of the superficial and deep heads of the PQ during grip strength [18]. Further studies have also identified the strong correlation with grip strength and pronation strength advocated for the need of normative values of pronation torque and grip strength in order to improve the quality of the clinical assessment of an injured wrist [18]. Testing these in our study identified similar traits as both outcomes were highly correlated with one another [19].

Grip strength was identified as being well correlated with pronation strength in both treated and non-treated forearms. In the literature, it is common for grip strength to be reduced in the initial 12 months following volar plating surgery when the PQ is not repaired. However, a meta-analysis by Stinton et al. has reported that grip strength may show significant change up 24 months post-surgery [20]. Our study showed a strong grip strength ratio (GSR). GSR is a quantitative measure identifying grip strength weakness between an injured and non-injured forearm. A low grip strength ratio is suggestive of high discrepancy between injured and non-injured, and the converse is true with a high grip strength ratio. In Breumur et al. [15], the GSR of a cohort of 20 healthy non-injured individuals was 0.97, and our results are comparable with a GSR of 0.98. It was not possible to compare the cohort of volar plated patients in Breumur et al. as there is no mention of the post-operative stages at which they assessed patients, nor whether the PQ was repaired. In a recent study by Goorens et al. (24), differences in grip strength and range of motion (ROM) were not found to be significant after 12 months in repaired vs non-repaired patients; however, pronation torque was not specifically measured. Hand dominance has been reported to effect grip strength by up to 10% in favour of the dominant hand. Our patient cohort was comprised of 13 dominant and 14 non-dominant forearms treated with volar plating. In the present study, hand dominance was not recorded as there was no difference between mean peak torque or grip strength.

### Study limitations

It has been reported in the literature for ROM and grip strength to return to normal at 12 months [20]. However, Ydreborg et al.’s prospective repeated-measure study identified patients reporting pain and disability at rest for up to 24 months [21]. This potential relationship between subjective (patient reported pain and disability) and objective measurements (ROM and grip strength) was not investigated in this study. In addition, a patient rated outcome measure (PROMS) was not used in line with Johnson et al.’s recommendation that there is insufficient recommendation of the optimal PROMS for patients [22]. Although this study has not shown any functional benefit of repairing the PQ, some surgeons recommend that it reduces the risk of flexor tendon rupture by acting as a physical barrier between the plate and the tendons. However, there is limited evidence of the effectiveness of this [23] and a further study into this topic would be beneficial.

## Conclusion

Our findings suggest that not repairing the PQ during volar plating will not lead to a functional diminution in pronation strength in patients reviewed at 12 months post-operatively. The addition of these results to the literature may inform surgical decision making: if volar plating is carried out and the PQ is released and not repaired, then it is not likely to cause functional reduction of forearm pronation. Eliminating the step of repairing the PQ muscle will save operative time, reducing the length of the anaesthetic and tourniquet for the patient. This study adds novel biomechanical information to the discussion of surgical fixation of the distal radius and offers a modification to the closure of the wrist incision.

## Data Availability

The datasets generated during and/or analysed during the current study are available from the corresponding author on reasonable request.

## Code availability

The code used during the current study is available from the corresponding author on reasonable request.
